# Dynamic changes of CSF clusterin levels across the Alzheimer’s disease continuum

**DOI:** 10.1186/s12883-022-03038-w

**Published:** 2022-12-30

**Authors:** Lian Tang, Zhi-Bo Wang, Ling-Zhi Ma, Xi-Peng Cao, Lan Tan, Meng-Shan Tan

**Affiliations:** 1 Department of Neurology, Qingdao Municipal Hospital, Qingdao University, Qingdao, China; 2 Clinical Research Center, Qingdao Municipal Hospital, Qingdao University, Qingdao, China

**Keywords:** Alzheimer’s disease, Aβ, Clusterin, CSF, Tau, Neurodegeneration

## Abstract

**Background:**

Clusterin is a multifunctional protein, which is associated with the pathogenesis and the development of Alzheimer’s disease (AD). Compared with normal controls, inconsistent results have yielded in previous studies for concentration of cerebrospinal fluid (CSF) clusterin in AD patients. We explored CSF clusterin levels in different pathological processes of AD.

**Methods:**

Following the National Institute on Aging-Alzheimer’s Association (NIA-AA) criteria, we employed on the levels of CSF Aβ_42_(A), phosphorylated-Tau (T), and total-tau (N). Based on previously published cutoffs and the close correlation between CSF p-tau and t-tau, 276 participants from the publicly available ADNI database with CSF biomarkers were divided into four groups: A-(TN)- (normal Aβ_42_ and normal p-tau and t-tau; *n* = 50), A+(TN)- (abnormal Aβ_42_ and normal p-tau and t-tau; *n* = 39), A+(TN) + (abnormal Aβ_42_ and abnormal p-tau or t-tau; *n* = 147), A-(TN) + (normal Aβ_42_ and abnormal p-tau or t-tau; *n* = 40). To assess CSF clusterin levels in AD continuum, intergroup differences in four groups were compared. Pairwise comparisons were conducted as appropriate followed by Bonferroni post hoc analyses. To further study the relationships between CSF clusterin levels and AD core pathological biomarkers, we employed multiple linear regression method in subgroups.

**Results:**

Compared with the A-(TN)- group, CSF clusterin levels were decreased in A+ (TN)- group (*P* = 0.002 after Bonferroni correction), but increased in the A+(TN) + group and the A-(TN) + group (both *P* <  0.001 after Bonferroni correction). Moreover, we found CSF clusterin levels are positively associated with CSF Aβ_42_ (β = 0.040, *P* <  0. 001), CSF p-tau (β = 0.325, *P* <  0.001) and CSF t-tau (β = 0.346, *P* <  0.001).

**Conclusions:**

Our results indicated that there are differences levels of CSF clusterin in different stages of AD pathology. The CSF clusterin level decreased at the early stage are related to abnormal Aβ pathology; and the increased levels are associated with tau pathology and neurodegeneration.

**Supplementary Information:**

The online version contains supplementary material available at 10.1186/s12883-022-03038-w.

## Background

Alzheimer’s disease (AD) is a devastating neurodegenerative disorder affecting in the world widely [1]. At the neuropathological level, in a specific temporal-ordered manner，the accumulation of AD pathology develops [2]. Up till now, the amyloid-β (Aβ) cascade hypothesis has been the most important model accounting for the pathogenesis of AD. The hypothesis proposed extracellular Aβ deposition occurred AD early pathology, leading to the deposition of intracellular phosphorylated tau and neurodegeneration [3].

Clusterin (CLU; also known as apolipoprotein J, ApoJ) is a multifunction protein widely expressed in vivo, attributing functions obtaining immune modulation, regulation Aβ metabolism, Aβ clearance in AD [4]. Primary structure of CLU is encoded by *CLU* gene which contains single nucleotide polymorphisms (SNP’s) associated with the risk of late-onset Alzheimer’s disease (LOAD) [5–7]. Accumulating evidence has indicated that elevated clusterin levels contributes to AD early pathogenesis (Desikan et al. 2014; Oh et al. 2019). While, research produced inconsistent results on the concentration of CSF clusterin in AD patients. Compared with healthy controls, some studies showed increased levels of CSF clusterin were in the AD patients [8–10].However, decreased levels of CSF clusterin were observed in some studies in AD patients [11, 12]. Recently, two meta-analyses showed that there were no differences in CSF clutserin levels between AD patients and normals [13, 14]. We hypothesized the inconsistency might be due to CSF clusterin levels were different during different AD pathological stages.

Studies have shown clusterin involved in AD pathology. Previous studies have shown that clusterin exerts protective effects in AD, including markedly improving Aβ clearance by binding to Aβ across the blood-brain barrier (BBB) [15], preventing Aβ aggregation [16, 17], and promoting lysosomes Aβ degradation [15, 18], which suggest clusterin was considered as a protector in AD. However, a recent study has shown that elevated levels of clusterin may accelerate the spreading of tau aggregates in AD patients, suggesting that clusterin can speed up AD progression [19]. Taken together, these findings imply that clusterin serves a complicated influence in the AD pathological process. The relationship between clusterin and AD may vary at the different stages of AD pathology. Therefore, our study aims to study CSF clusterin levels in different pathological stages of AD.

We employed the ATN classification system based on the CSF levels of amyloid-β (1–42) (A), phosphorylated-Tau (T), and total-tau (N) [20], which is proposed by the 2018 National Institute on Aging-Alzheimer’s Association (NIA-AA) Research Framework [21]. We dichotomized biomarkers according to use previously published cutoffs as normal (negative) or abnormal (positive). As previously described [21], eight ATN biomarker profiles were originally created. However, our data is relatively small for some profiles. To define groups with more samples that are adequate for analysis, our research divided ADNI participants into four groups by four different AD pathological stages and merged T and N as TN, given CSF p-tau and t-tau are closely related. Thus, four groups were obtained, including the normal AD biomarkers groups(A-(TN)-), only Aβ-pathology groups (A+(TN)-), both Aβ-pathology and its downstream processes of tau pathology or neurodegeneration A+(TN)+, and suspected non-AD pathology groups (A-(TN)+). Our study firstly aims at exploring the CSF clusterin levels in different pathological stages, and secondly investigating the correlation of CSF clusterin levels with core pathological AD biomarkers in the biomarker normal group and AD continuum group.

## Methods

### ADNI database

This study was based on publicly available data from the Alzheimer’s Disease Neuroimaging Initiative (ADNI) database. ADNI aims to examine whether clinical, imaging and genetic assessments as well as CSF biomarkers can be integrated to detect and track AD as soon as possible. Any neurological disease patients were excluded from the database, except suspected AD. The ages of participants are in 55 to 90. On the ADNI website, more detailed information about inclusive or exclusive criteria can be found. In accordance with the Declaration of Helsinki, written informed consent was acquired from all the participants or their representatives. ADNI was approved by the institutional review boards of all the participating institutions. For latest information, see ADNI website. [22, 23].

### Participants

We included 285 participants with baseline data on CSF clusterin, CSF amyloid-β (1–42) (Aβ_42_), phosphorylated-Tau (p-tau), and total-tau (t-tau) levels from the ADNI database. Nine participants with values outside the mean ± 3 standard deviations (SD) were excluded, leaving 276 participants for further analysis.

### Measurements of CSF biomarkers

CSF Aβ_42_, p-tau, t-tau were all analyzed using the INNO-BIA Alz-Bio3 immunoassay. These within-batch precision values were all below 10% (respectively Aβ_42_: 5.1 to 7.8%, p-tau: 5.1 to 8.8%, t-tau: 4 to 9.8%). At the same time, these CSF core biomarkers were analyzed by the fully automated Elecsys immune assay platform® at the University of Pennsylvania. More detailed information about Elecsys method measuring AD biomarkers in ADNI is provided elsewhere [24]. All CSF biomarkers assays were repeated and averaged. The quantification of CSF clusterin was conducted using liquid chromatography-tandem mass spectrometry with multiple reaction monitoring approach (LC/MS-MRM) [25]. The whole panel consist of 567 petides representing 221 proteins, and the peptide sequence used for clusterin was IDSLLENDR in our research. We obtained data from ADNI at http://adni.loni.usc.edu/.

### ATN classification system

Following 2018 NIA-AA criteria, each ADNI participant was assigned into groups by the ATN framework [21]. At present, this classification system serves research purposes rather than clinical purposes. Additionally, ATN classification system is useful for observational studies since it stresses the predictive value of biomarkers [26]. The primary criteria for AD classification are dependent on underlying pathophysiological damage rather than clinical manifestations. In the ATN classification system, “A” refers to aggregated Aβ (CSF Aβ_42_ or amyloid PET); “T” refers to aggregated tau (CSF p-tau or tau PET); and “N” refers to neurodegeneration (CSF t-tau, FDG-PET or MRI) [21, 27]. In our study, we implemented this classification based on CSF Aβ_42_ levels (A); CSF p-tau levels (T); CSF t-tau (N). To reduce comparison groups numbers, the tau pathology group (T) and neurodegeneration group (N) were merged, given that CSF p-tau and t-tau are closely correlated. ‘(TN)-’ profile was defined as both normal range of CSF p-tau and t-tau and ‘(TN)+’ profile was defined as p-tau or t-tau with abnormal range. We dichotomized biomarkers according to use previously published cutoffs as normal (negative) or abnormal (positive). CSF Aβ_42_ < 976.6 pg/mL, CSF p-tau > 21.8 pg/mL, CSF t-tau > 245 pg/ml were respectively defined as A positive (A+), T positive (T+) and N positive (N+) [28]. Then, we obtained four different groups, containing A–(TN)–, A+(TN)–, A+(TN)+, A-(TN)+. Specifically, participants with normal pathology defined as “A–(TN)–”, abnormal amyloid with normal t-tau and p-tau defined as “A+(TN)–”, abnormal amyloid and abnormal levels of t-tau/p-tau defined as “A+(TN)+”, and suspected non-AD pathology defined as A-(TN)+. In addition, individuals were divided into the biomarker normal group (A–(TN)–) and AD continuum group (A+(TN)– and A+(TN)+).

### Statistical analysis

The data downloaded from the ADNI database showed that the concentration of CSF clusterin displayed an approximate normal distribution, which was presented in the Q-Q plot (Supplementary Fig. 1). CSF Aβ_42_, CSF p-tau and t-tau levels were log-transformed to obtain normal distributions. We excluded values outside the mean ± 3 standard deviations (SD) to eliminate the influence of extreme values. Totally, nine individuals were excluded in this step. To explore the differences in CSF biomarkers and sociodemographic data, we employed one-way analyses of variance (ANOVA) for continuous variables and chi square for dichotomous variables (such as *APOE ε4* status and gender). Subsequently, to test the levels of CSF clusterin in different ATN groups, we employed an analysis of covariance (ANCOVA) followed by Bonferroni post hoc analyses. Thus, the threshold *P* values of groups differences for statistical significance was set at 0.008 (= 0.05/6). Finally, in the whole cohort, the biomarker normal group and the AD continuum group, a multiple linear regression was performed to study the associations of the concentration of CSF clusterin with CSF core pathological biomarkers, adjusting for age, diagnosis, gender, education, and *APOE ε4* status. *P* <  0.05 was defined as statistical significance the correlation analysis. We used R software version 4.1.0 and IBM SPSS Statistics to perform all statistical analyses.

## Results

### Subjects’ characteristics

The demographics and clinical features of participants at baseline are summarized in the Table 1. Our current study, 276 participants were included from the publicly available ADNI database (A-(TN)-, *n* = 50; A+(TN)-, *n* = 39; A+(TN)+, *n* = 147; A-(TN)+, *n* = 40). The study population had a female population of 39.5%, an *APOE ε4* positive percentage of 47.8%, an average age of 75.11 ± 6.86 years, and 15.72 ± 2.99 years of education. Four groups showed statistically significant differences in the distribution of *APOE ε4* status and MMSE score (both *P* <  0.001) rather than age, education level, and gender. The proportion of MMSE scores and *APOE ε4* are dependent on the ATN framework, of which the highest percentage of *APOE ε4* and lowest MMSE scores are in A+ profiles. Compared with the other three groups, A+(TN)- group showed the lowest levels of CSF clusterin.

**Table 1:** Characteristics of participants based on ATN classification

Characteristics	A-(TN)-	A+(TN)-	A+(TN)+	A-(TN)+	***P***
N	50	39	147	40	
Clinical diagnosis details (n, %)					
CU	37(44.05)	13(15.48)	16(19.04)	18(21.43)	
MCI	11(9.40)	6(5.13)	82(70.09)	18(15.38)	
AD	2(2.67)	20(26.67)	49(65.33)	4(5.33)	
Age (Mean ± SD, years)	74.53 ± 6.09	75.58 ± 5.23	74.66 ± 7.26	77.00 ± 7.46	0.232^a^
Gender (F/M)	19/31	12/27	61/86	17/23	1.000^b^
Education (Mean ± SD, years)	15.28 ± 3.01	15.51 ± 3.50	15.83 ± 2.93	16.10 ± 2.62	0.514^a^
*APOE ε4* (n, %)	3 (6.00)	20 (51.28)	102 (69.39)	7 (17.50)	< 0.001^b^
MMSE score	28.44 ± 1.47	26.51 ± 2.45	25.69 ± 3.31	28.20 ± 1.74	< 0.001^a^
CSF biomarker					
Aβ_42_ (pg/ml)	1441.64 ± 252.09	639.87 ± 194.49	587.76 ± 176.21	1794.03 ± 664.20	< 0.001^a^
t-tau (pg/ml)	191.10 ± 31.10	181.61 ± 33.97	353.71 ± 97.99	320.71 ± 81.04	< 0.001^a^
p-tau (pg/ml)	16.78 ± 2.87	16.66 ± 3.53	35.99 ± 10.63	29.09 ± 9.66	< 0.001^a^
Clusterin	20.56 ± 0.45	20.24 ± 0.45	20.38 ± 2.02	20.98 ± 0.36	< 0.001^a^

Numbers and percentages are used to express categorical variables. Means ± SDs is used to express continuous variables.

Abbreviations: *CU* Cognitively unimpaired, *MCI* Mild cognitive impairment, *AD* Alzheimer’s disease, *F* Female, *M* Male, *APOE* Apolipoprotein E, *SD* Standard deviations, *MMSE* Mini Mental State Examination, *CSF* cerebrospinal fluid, *Aβ*_*42*_ amyloid-β 42, *p-tau* phosphorylated-tau, *t-tau* total-tau

^a^ Comparison of subgroups were employed by analyses of variance

^b^ Comparison of subgroups were employed by chi square

### Differences in CSF clusterin across the AD continuum

**Fig. 1:**
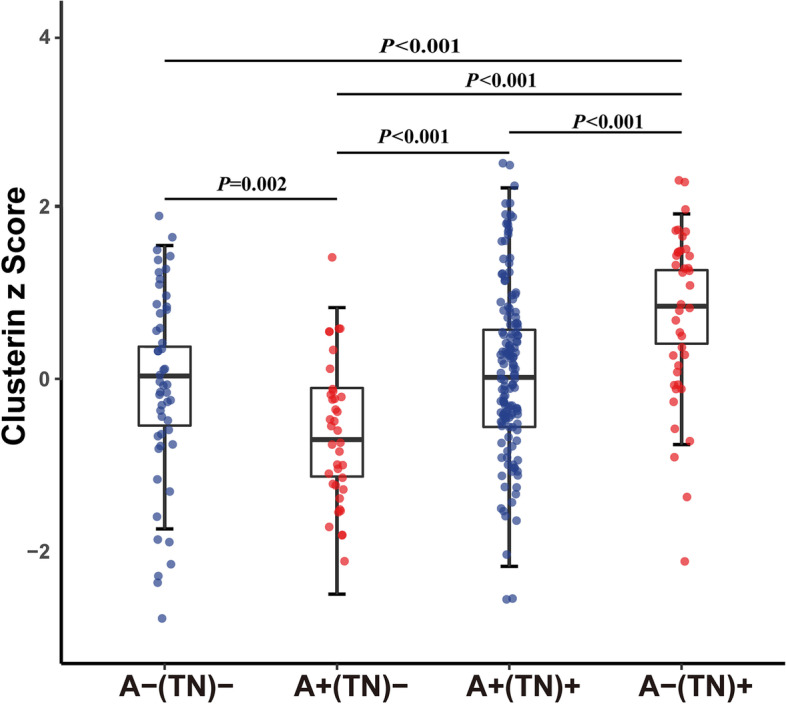
CSF clusterin in the ATN classification. The levels of CSF clusterin in the four biomarker profiles were described by scatter plots. The difference in four groups employed an analysis of covariance (ANCOVA) followed by Bonferroni post hoc analyses. The significant *P*-values were marked after Bonferroni correction

To assess the levels of CSF clusterin across the AD continuum, we employed ATN classification framework. Significant differences were shown by one-way ANCOVA after Bonferroni correction among the four groups (Fig. 1). As mentioned above, the A+(TN)- group had the lowest concentration of CSF clusterin. And compared to the A+(TN)- group, increased level of CSF clusterin was in the A+(TN) + group (*P* <  0.001 after Bonferroni correction). Considering age, gender, *APOE ε4*, education level all influence AD, we conducted another ANCOVA after adjusting for all these factors, which yielded the same results as previous studies.

We also repeated ANCOVA in groups classified by Aβ pathology and tau pathology status or in groups classified by Aβ pathology and neurodegeneration status. The results of the two analyses are presented in the Supplementary Fig. 3 and Supplementary Fig. 4, which are consistent with those based on ATN classification system. This consistency suggests that the preceding grouping is reasonable.

Our results suggest that during AD progression, CSF clusterin levels are different, which may be associated with the pathological changes in ATN biomarkers.

### CSF clusterin levels in the A-(TN) + group

Participants who had abnormal tau pathology or neurodegeneration without amyloidosis(A-(TN) + group)were considered as suspected AD. The results of the Bonferroni post hoc test showed CSF clusterin levels were different among groups. As shown in Fig. 1, the higher level of CSF clusterin was in the A-(TN) + group compared to the A-(TN)- group (no pathology, *P* <  0.001 after Bonferroni correction), the A+(TN)- group (amyloid-only pathology, *P* <  0.001 after Bonferroni correction), and the A+(TN) + group (abnormal amyloid and abnormal t-tau/p-tau, *P* <  0.001 after Bonferroni correction) after adjusting for age, diagnosis, education, gender, and *APOE ε4* status. This result suggests that the elevation of CSF clusterin level may be associated with tau pathology/neurodegeneration.

### CSF clusterin and AD core biomarkers

**Fig. 2:**
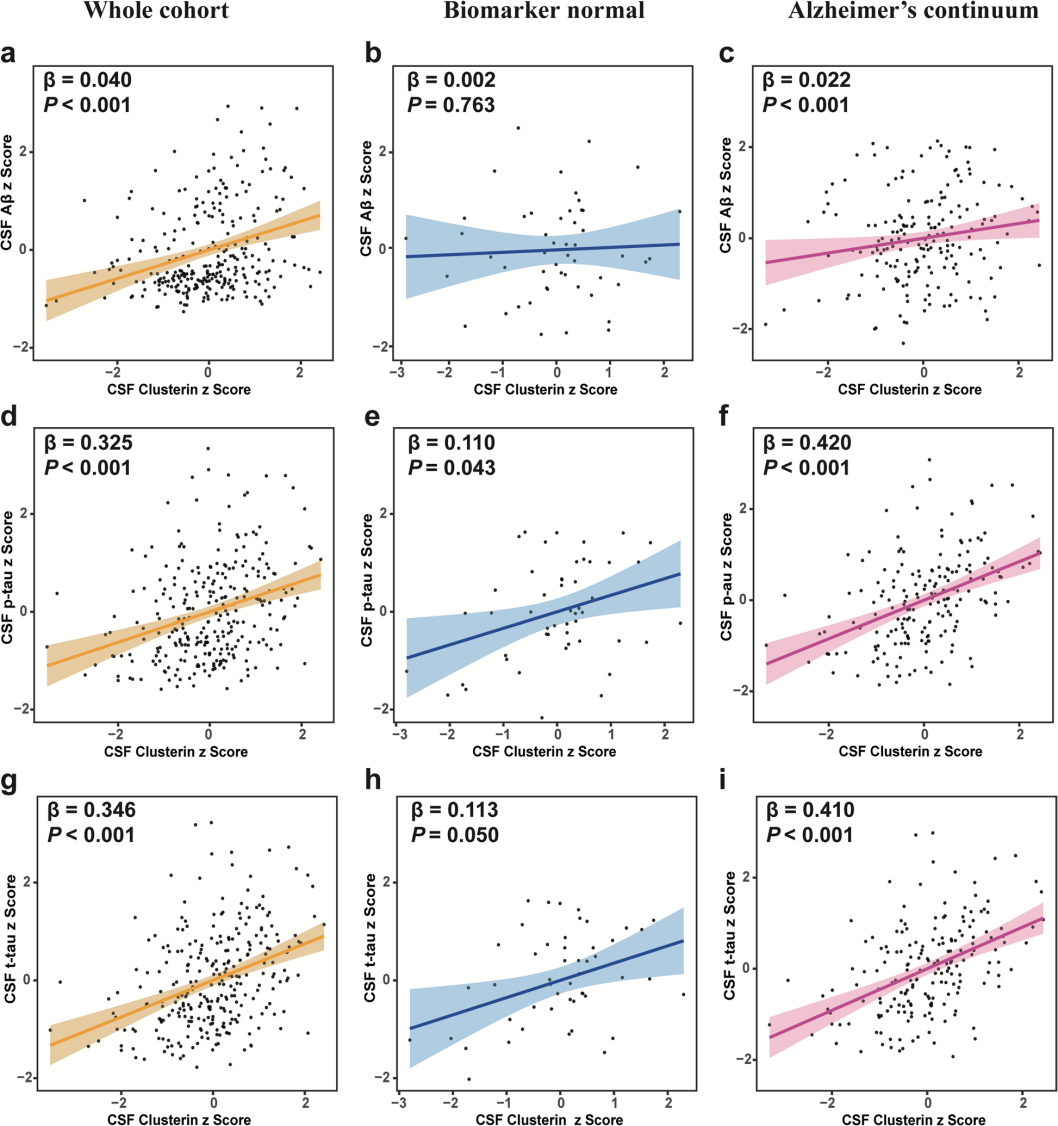
Associations of CSF clusterin levels with AD core pathological biomarkers. Scatter plots depict the associations of CSF clusterin with core pathological biomarkers: Aβ_42_, p-tau and t-tau in whole cohort (**a, d, g**), biomarker normal (**b, e, h**) and Alzheimer’s continuum (**c, f, i**). The multiple linear regression was applied to compute the standardized regression coefficients (β) and the *P*-values, containing age, diagnosis, gender, education, and *APOE ε4* status as covariates

Finally, to study the associations between CSF clusterin and the core AD biomarkers based on ATN system, we employed the linear regression methods adjusting for age, diagnosis, education, gender, and *APOE ε4* status. We excluded participants in the A-(TN) + group in this analysis. These results were presented in Supplementary Table 1. In all the participants (*n* = 237), CSF clusterin was positively associated with CSF Aβ_42_ (β = 0.040, *P* <  0.001) (Fig. 2a), CSF p-tau (β = 0.325, *P* <  0.001) (Fig. 2d), and CSF t-tau (β = 0.346, *P* < 0.001) (Fig. 2g). We further investigated these correlations in the subgroups, including biomarker normal and AD continuum groups. Figure 2b showed no significant association was observed between CSF clusterin and CSF Aβ_42_ in the biomarker normal group (β = 0.002, *P* = 0.763). In AD continuum groups, there was a positive relationship between CSF clusterin and CSF Aβ_42_ in Fig. 2c. Results showed the CSF clusterin were positively associated with CSF t-tau and CSF p-tau both in biomarker normal group and AD continuum group (Fig. 2h, e, i, f). We studied the association of CSF clusterin with CSF Aβ_42_ among A-TN- and A + TN- subjects. There was a positive relationship between CSF clusterin and CSF Aβ_42_ (β = 0.026, *P* < 0.005). We repeated the analysis after excluding the outliers, which yielded the similar results Supplementary Table 2.

## Discussion

In our study, we assessed the CSF clusterin levels across AD continuum using the ATN classification system. We further explored the associations of CSF clusterin with AD core pathological biomarkers in different subgroups. Our results indicated that CSF clusterin levels were different across AD continuum influenced AD pathologies: the decreased levels of CSF clusterin at early stages are related to abnormal Aβ pathology; and the elevated levels of CSF clusterin are related to tau pathology and neurodegeneration.

Our data proved our hypothesis that the levels of CSF clusterin have different levels across the AD continuum rather than a unidirectional increase or decrease as shown by previous studies. The different levels of clusterin during different AD stages have not been observed by previous studies due to their adoption of clinical staging. Compared to a previous ADNI study with clusterin increasingly in AD patients [10], our study provides more samples and extends into the ATN system. Since we used ATN classification, different levels of CSF clusterin during different AD pathological stages became evident across AD severity spectrum. We found that decreased level of CSF clusterin was initially observed in amyloid-only pathology, which was considered as the earliest stage of AD, and elevated levels of CSF clusterin were observed in downstream tau pathology and neurodegeneration. In light of previous studies, the mechanisms underlying the different levels of CSF clusterin might be as follows. As for the lowered CSF clusterin levels in amyloid-only pathology, one probably explanation is that CSF clusterin can bind with Aβ and produce complexes [29], which may lead to lower CSF clusterin; another potential explanation is that Aβ can promote degradation of clusterin lysosomes via the expression of sortilin in the early stage of AD [30]. As for the increased CSF clusterin levels in tau pathology and neurodegeneration, the explanation may be that clusterin increases in response to inflammatory processes mediated by cytokine in plaques [31, 32]. Our finding was in line with those of previous studies showing that this increase in CSF clusterin levels was a response to Aβ stress and neural injury [33, 34]. CSF clusterin will be a promising candidate marker to reflect disease progression and be a potential outcome parameter in future trials.

In the present study, compared to the A-(TN)- group, higher levels of CSF clusterin were observed in both A+(TN) + and A-(TN) + groups, suggesting that increases in CSF clusterin levels were related to tau pathology or neurodegeneration. These results are consistent with that of a recent study which indicated that increased clusterin levels occurred not only in the presence of amyloid plaques but also the primary tauopathy [35]. The cause-and-effect relationship between increased CSF clusterin and tau pathology/neurodegeneration is ambiguous. Some studies showed that AD patients had increased clusterin levels due to neurodegeneration, which could significantly ameliorate tau pathology by inhibiting fibril formation (Nuutinen et al. 2009; Schrijvers et al. 2011; Cunin et al. 2016). However, a previous animal study found that an exogenous injection of clusterin could increase the levels of tau proteins, suggesting clusterin could aggravate tau pathology (Martin-Rehrmann et al. 2005).

We further investigated the associations between CSF clusterin and CSF core biomarkers separately in total individuals, biomarker normal, and AD continuum group. Both in total participants and AD continuum group, CSF clusterin levels were positively related to CSF Aβ_42_, CSF p-tau, and CSF t-tau, which was consistent with most previous studies. Clusterin and p-tau/t-tau might be linked via neuronal damage [36]. Interestingly, CSF clusterin was also positively associated with CSF t-tau and p-tau in biomarker normal. This might be explained by physiological aging processes, considering the close relationship between age and clusterin in Supplementary Fig. 2. Previous studies have showed clusterin may be a protective participant in, such as adaptive responses to regeneration of mild injured neurons and neuroinflammation [37–39].

Previous studies did not focus on levels of CSF clusterin in different stages of AD. The highlight of the current research is that it is the first to study the CSF clusterin levels in different AD pathological stages. And an AD biomarker classification system we used based on the diagnostic guidelines of the NIA-AA study. There is a temporal sequence on the models of AD pathophysiology theorize. Extracellular Aβ deposition(A) initiates a biological cascade, followed by phosphorylated tau aggregation (T) that leads to neurodegeneration (N) [3, 40]. Based on the classical A-T-N sequence, we suspect that CSF clusterin levels may dynamically change, associated with the pathological changes in ATN biomarkers. Nevertheless, we recognize that our research is only at the level of observation and limits any conclusion about disease progression. Further studies are required more follow-up data to study the dynamic evolution of clusterin in AD pathology. Also, more animal experiments are needed to uncover the underlying mechanisms in the dynamic CSF clusterin in disease progression.

## Conclusion

In the current research, we assessed CSF clusterin levels in the Alzheimer’s continuum based on ATN system classification. The application of this system made us unravel that CSF clusterin levels are different in the development of the pathological stage in AD. To sum up, the decreased levels of CSF clusterin at an early stage are related to abnormal Aβ pathology; and the enhanced levels of CSF clusterin are related to tau pathology and neurodegeneration. Our study provided underlying simultaneous processes in AD progression. Future studies should use longitudinal data and explore the underlying mechanisms in the dynamic CSF clusterin.

## Supplementary Information

**Additional file 1: Supplementary Fig. 1.** CSF clusterin normal Quantile-Quantile Plot, **Supplementary Fig. 2.** Association of CSF clusterin with age, **Supplementary Fig. 3.** CSF clusterin in groups defined only by CSF Aβ_42_ and p-tau, **Supplementary Fig. 4.** CSF clusterin in groups defined only by CSF Aβ_42_ and t-tau, **Supplementary Table 1.** Association of CSF clusterin and CSF core biomarkers, **Supplementary Table 2.** Association of CSF clusterin and CSF core biomarkers containing outliers.

## Data Availability

The data used in preparation for this article were obtained from the publicly available ADNI database (https://adni.loni.usc.edu/). The datasets used and/or analyzed during the current study are available from the corresponding author on reasonable request.

## Abbreviations

CSF: cerebrospinal fluid
AD: Alzheimer’s disease
NIA-AA: National Institute on Aging–Alzheimer’s Association
F: Female
M: Male
APOE: Apolipoprotein E
SD: Standard deviations
MMSE: Mini Mental State Examination
Aβ_42_: amyloid-β 42
p-tau: phosphorylated-tau
t-tau: total-tau
ANCOVA: analyses of variance
A-: cerebrospinal fluid normal
A +: cerebrospinal fluid amyloid-β 42 below the reference range
T-: cerebrospinal fluid phosphorylated-tau normal
T +: cerebrospinal fluid phosphorylated-tau above the reference range
N-: cerebrospinal fluid total-tau normal
N +: cerebrospinal fluid total-tau above the reference range
(TN)-: cerebrospinal fluid phosphorylated-tau and total-tau normal
(TN) +: cerebrospinal fluid phosphorylated-tau above or total-tau above the reference range
