# Supplementary material for: Dynamic changes of CSF clusterin levels across the Alzheimer’s disease continuum
Source: BMC Neurol. 2022 Dec 30;22:508. doi: 10.1186/s12883-022-03038-w (PMC9801612; doi:10.1186/s12883-022-03038-w)
Supplement: Supplementary file 1 — Additional file 1: Supplementary Fig. 1. CSF clusterin normal Quantile-Quantile Plot, Supplementary Fig. 2. Association of CSF clusterin with age, Supplementary Fig. 3. CSF clusterin in groups defined only by CSF Aβ42 and p-tau, Supplementary Fig. 4. CSF clusterin in groups defined only by CSF Aβ42 and t-tau, Supplementary Table 1. Association of CSF clusterin and CSF core biomarkers, Supplementary Table 2. Association of CSF clusterin and CSF core biomarkers containing outliers. [file 12883_2022_3038_MOESM1_ESM.docx]

# Contents

# Supplementary Fig.1 CSF clusterin normal Quantile-Quantile Plot

# Supplementary Fig.2 Association of CSF clusterin with age

# Supplementary Fig.3 CSF clusterin in groups defined only by CSF Aβ_42_ and p-tau

# Supplementary Fig.4 CSF clusterin in groups defined only by CSF Aβ_42_ and t-tau

# Supplementary Table 1 Association of CSF clusterin and CSF core biomarkers

# Supplementary Table 2 Association of CSF clusterin and CSF core biomarkers containing outliers.


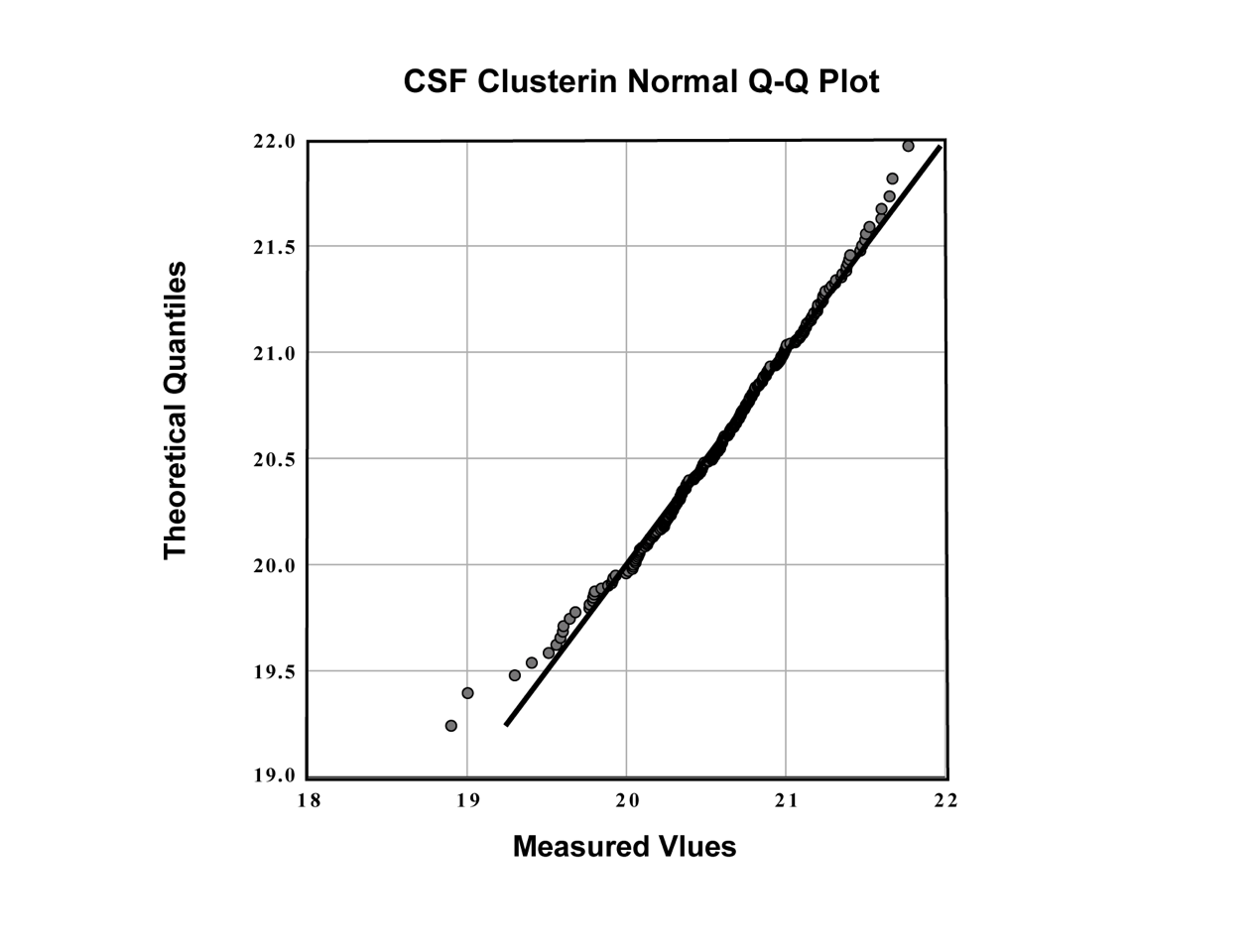


**Supplementary Fig.1 CSF clusterin normal Quantile-Quantile Plot.**

The actual data is on the X-axis, and the assumed normal data is on the Y-axis

Q-Q plot showed a normal distribution of CSF clusterin.

Abbreviations: CSF, cerebrospinal fluid.


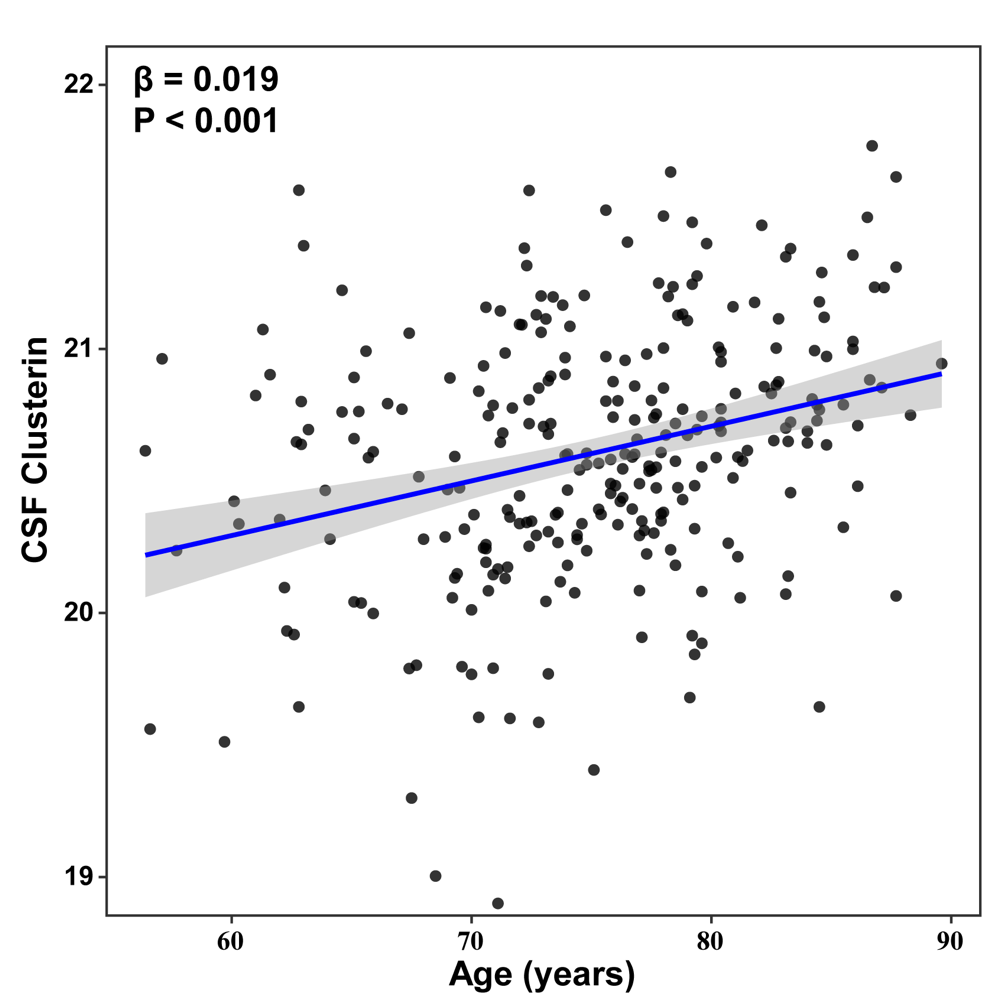


**Supplementary Fig.2 Association of CSF clusterin with age.**

The relationship between CSF clusterin and age was conducted by multiple linear regression.

Abbreviations: CSF, cerebrospinal fluid.


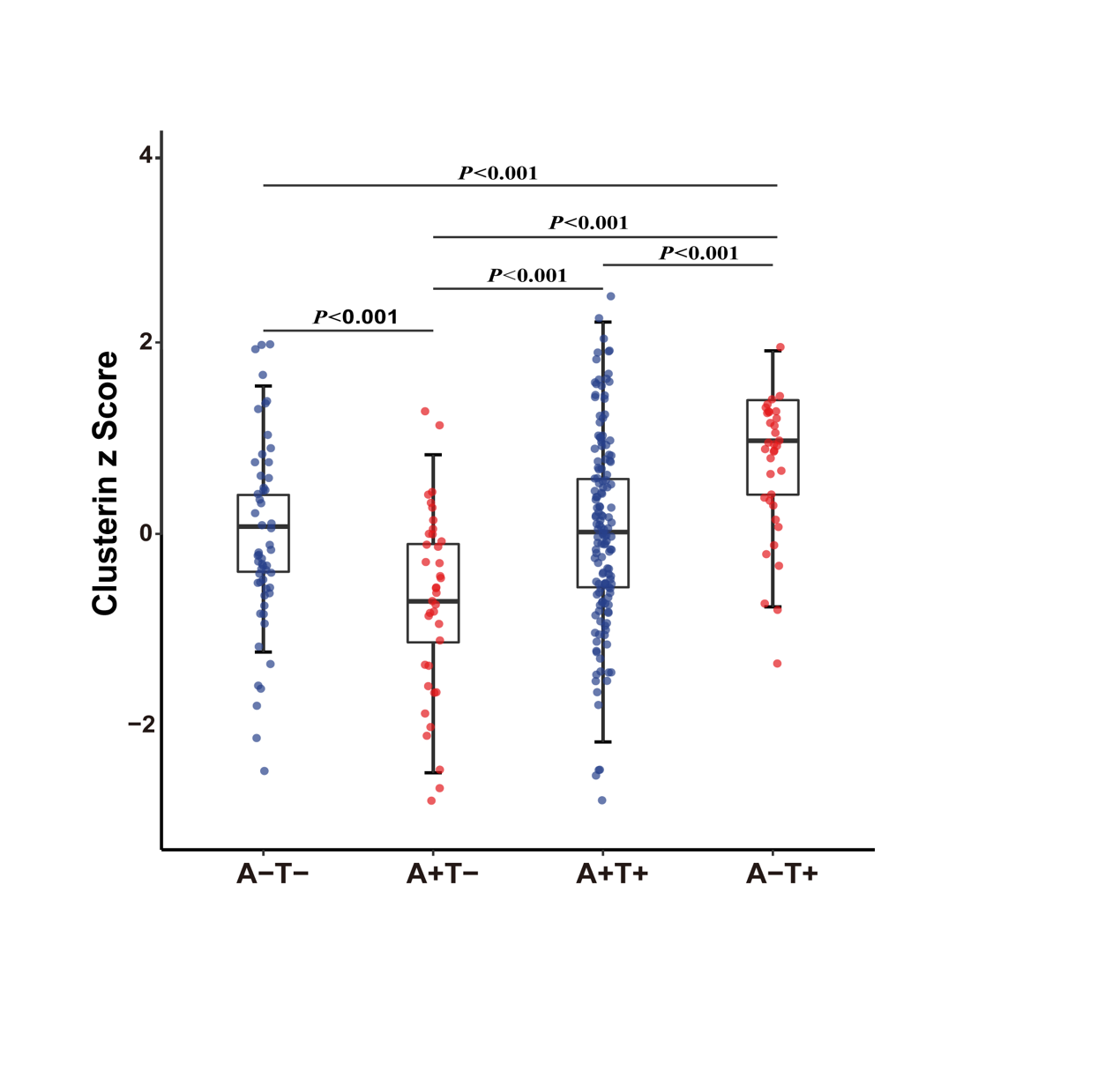


**Supplementary Fig. 3 CSF clusterin in groups defined only by CSF Aβ_42_ and p-tau.**

CSF clusterin in groups defined as CSF Aβ_42_ and p-tau. Based on the levels of CSF Aβ_42_ and p-tau, participants were divided four groups. The levels of CSF clusterin for each of the four biomarker profiles was described by scatter plots. Four groups of difference employed an analysis of covariance (ANCOVA) followed by Bonferroni post hoc analyses. The significant *P*-values were marked after Bonferroni post.

Abbreviations: CSF, cerebrospinal fluid; Aβ_42_, amyloid-β 42; p-tau, phosphorylated-tau; A-: cerebrospinal fluid amyloid-β 42 normal; A+: cerebrospinal fluid amyloid-β 42 below the reference range; T-: cerebrospinal fluid phosphorylated-tau normal; T+: cerebrospinal fluid phosphorylated-tau above the reference range.


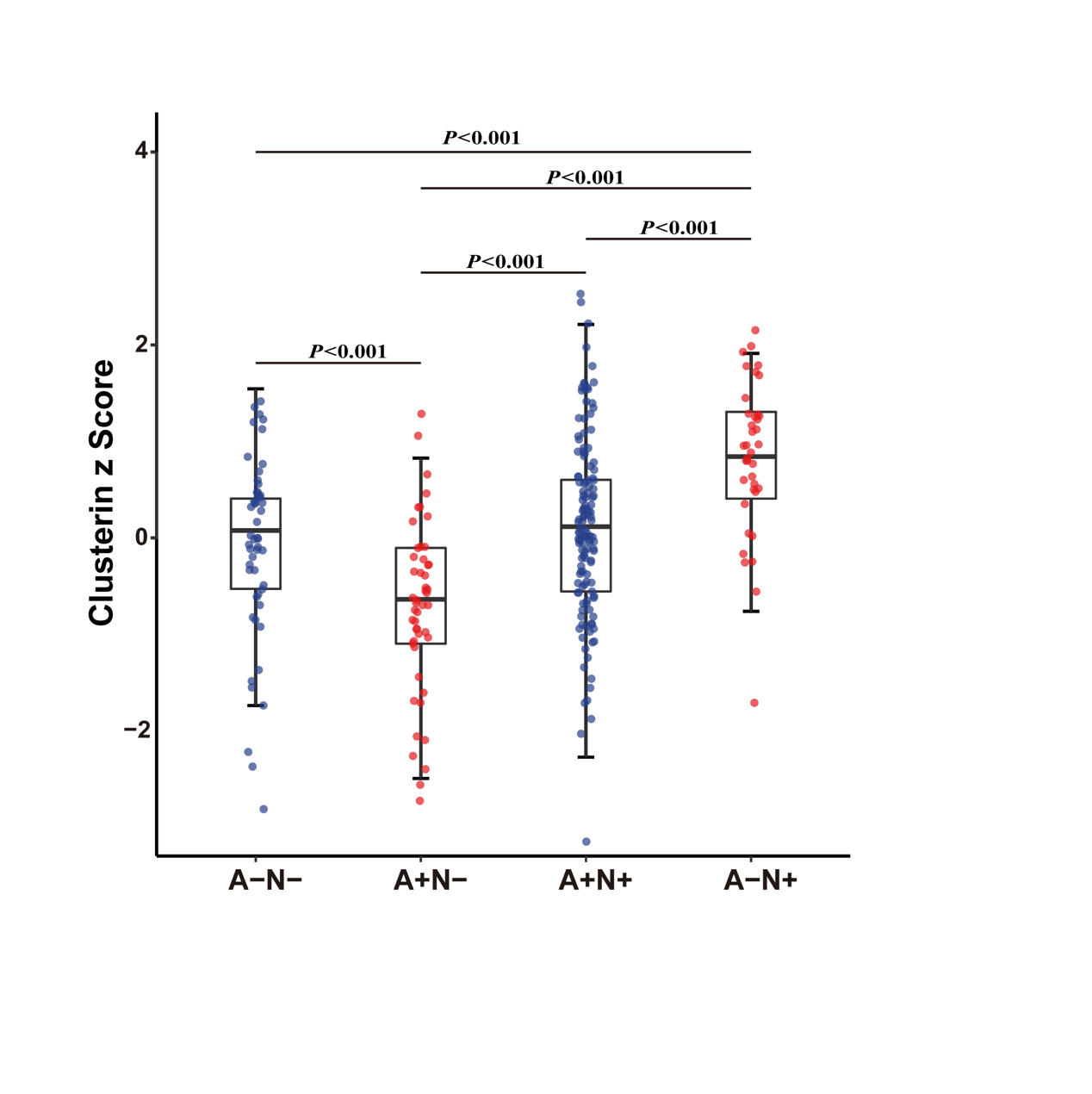


**Supplementary Fig. 4 CSF clusterin in groups defined only by CSF Aβ_42_ and t-tau.**

CSF clusterin in groups defined as CSF Aβ_42_ and t-tau. Based on the levels of CSF Aβ_42_ and t-tau, participants were divided four groups. The levels of CSF clusterin for each of the four biomarker profiles was described by scatter plots. Four groups of difference employed an analysis of covariance (ANCOVA) followed by Bonferroni post hoc analyses. The significant *P*-values were marked Bonferroni post.

Abbreviations: CSF, cerebrospinal fluid; Aβ_42_, amyloid-β 42; t-tau, total-tau; A-: cerebrospinal fluid amyloid-β 42 normal; A+: CSF Aβ_42_ below the reference range; N-: cerebrospinal fluid total-tau normal; N+: cerebrospinal fluid total-tau above the reference range.

**Supplementary Table 1 Association of CSF clusterin and CSF core biomarkers.**

| **CSF biomarker** | **Whole cohort** | |  | **Biomarker normal** | |  | **Alzheimer’s continuum** | |
| --- | --- | --- | --- | --- | --- | --- | --- | --- |
|  | **β** | ***P*** |  | **β** | ***P*** |  | **β** | ***P*** |
| Aβ_42_ | 0.040 | **<0.001** |  | 0.002 | 0.763 |  | 0.002 | **<0.001** |
| t-tau | 0.346 | **<0.001** |  | 0.113 | **0.050** |  | 0.410 | **<0.001** |
| p-tau | 0.325 | **<0.001** |  | 0.110 | **0.043** |  | 0.420 | **<0.001** |

The table showed adjusted *P*-values.

The age, gender, diagnose, education and *APOE ε4* status were adjusted.

Abbreviations: CSF, cerebrospinal fluid; Aβ_42_, amyloid-β 42; p-tau, phosphorylated-tau; t-tau, total-tau.

**Supplementary Table 2 Association of CSF clusterin and CSF core biomarkers containing outliers.**

| **CSF biomarker** | **Whole cohort** | |  | **Biomarker normal** | |  | **Alzheimer’s continuum** | |
| --- | --- | --- | --- | --- | --- | --- | --- | --- |
|  | **β** | ***P*** |  | **β** | ***P*** |  | **β** | ***P*** |
| Aβ_42_ | 0.012 | **<0.001** |  | 0.001 | 0.763 |  | 0.001 | **<0.001** |
| t-tau | 0.349 | **<0.001** |  | 0.113 | **0.050** |  | 0.395 | **<0.001** |
| p-tau | 0.338 | **<0.001** |  | 0.110 | **0.043** |  | 0.409 | **<0.001** |

The table showed adjusted *P*-values.

The age, gender, diagnose, education and *APOE ε4* status were adjusted.

Abbreviations: CSF, cerebrospinal fluid; Aβ_42_, amyloid-β 42; p-tau, phosphorylated-tau; t-tau, total-tau.
